# Cardiovascular Considerations in Patients Undergoing Hematopoietic Cell Transplantation

**DOI:** 10.1007/s11864-024-01240-1

**Published:** 2024-07-25

**Authors:** Jasmin Hundal, Thomas Curley, Betty K. Hamilton

**Affiliations:** 1 Hematology and Medical Oncology, Taussig Cancer Institute, Cleveland Clinic, Cleveland, OH USA; 2 Blood and Marrow Transplant Program, Hematology and Medical Oncology, Taussig Cancer Institute, 9500 Euclid Ave CA60, Cleveland, OH 44195 USA

**Keywords:** Cardiovascular disease, Hematopoietic cell transplant, Cardiac risk assessment

## Abstract

Cardiac dysfunction is a serious adverse effect of cancer therapies that can interfere with quality of life and impact long-term survival in patients with cancer. Hematopoietic cell transplantation is a potentially curative therapy for many advanced hematologic malignancies and bone marrow failure syndromes, however is associated with several short- and long-term adverse effects, including importantly, cardiovascular toxicities. The goal of this review article is to describe the cardiovascular events that may develop before, during, and after hematopoietic cell transplantation, review risk factors for short- and long-term cardiovascular toxicities, discuss approaches to cardiovascular risk stratification and evaluation, and highlight the research gaps in the consideration of cardiovascular disease in patients undergoing hematopoietic cell transplantation. Further understanding of cardiovascular events and the factors associated with cardiovascular disease will hopefully lead to novel interventions in managing and mitigating the significant long-term burden of late cardiovascular effects in transplant survivors.

## Introduction

Hematopoietic Cell Transplantation (HCT) is a potentially curative therapy for treating high-risk hematologic malignancies and bone marrow failure syndromes. Data from the Center for International Blood and Marrow Transplant Research (CIBMTR) report approximately 25,000 patients in the United States and 60,000 patients worldwide receive HCT annually [1]. With advancements in transplant approaches and supportive care, the number of transplant survivors continues to increase, estimated to be over 500,000 by 2030 [2]. In long-term survivors of transplant, mortality remains significantly elevated compared to the general population, even beyond 15 years, and cardiovascular disease (CVD) accounts for an increasing percentage of death [3]. Several studies have demonstrated an increased risk of CVD-related complications and a risk of CV death in HCT recipients 1.7–2.3 times that of the general population [4]. These findings highlight the need for better recognition and identification of high-risk patients for intervention in the prevention and treatment of CV complications in HCT recipients.

## HCT Overview

**Fig. 1:**
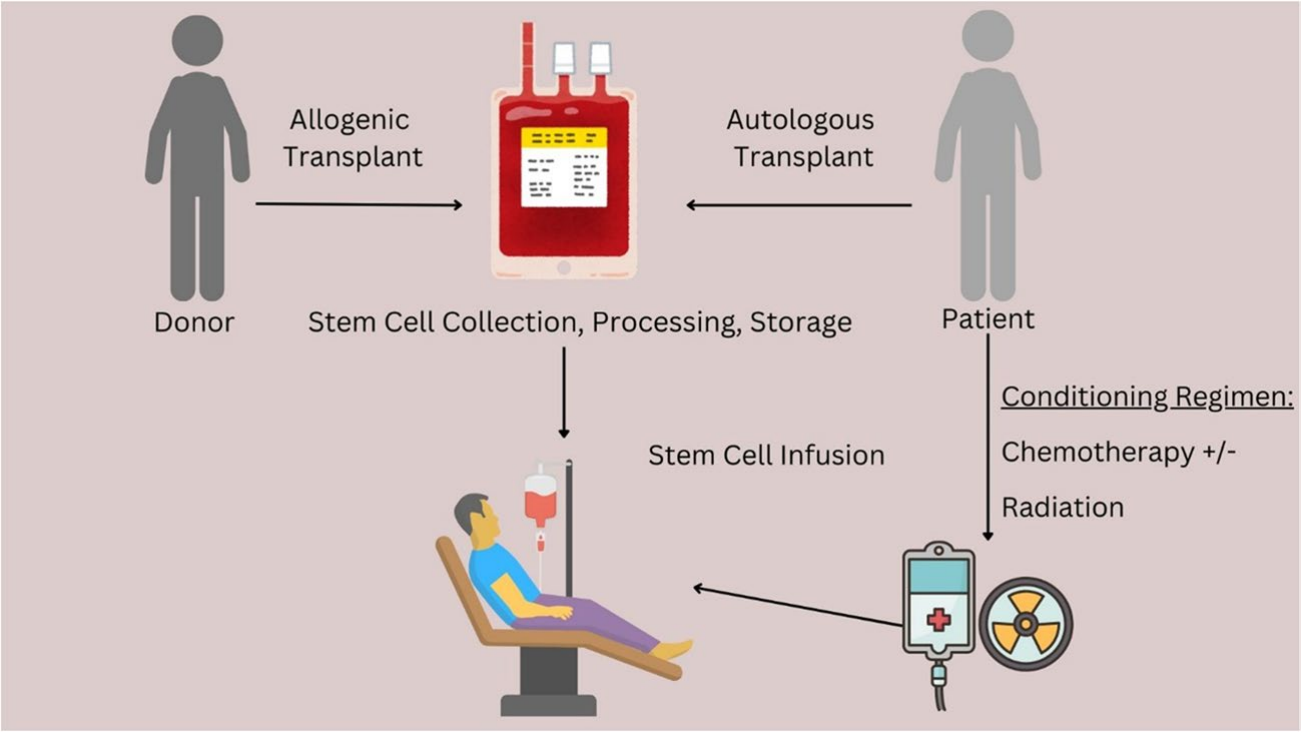
Hematopoietic Cell Transplantation (HCT) involves the administration of high doses of chemotherapy and/or radiation, followed by the infusion of hematopoietic progenitor cells. These cells can be sourced from the patient (autologous HCT) or a related or unrelated donor (allogeneic HCT)

HCT involves the administration of high doses of chemotherapy and/or radiation followed by the infusion of hematopoietic progenitor cells from the patient (autologous HCT) or a related or unrelated donor (allogeneic HCT) (Fig. 1). The rationale behind autologous HCT is to deliver sufficiently high doses of chemotherapy to eradicate tumor cells [5]. The subsequent infusion of autologous progenitor cells is to provide support for hematopoietic recovery. Allogeneic HCT is distinct from autologous HSCT as this procedure relies on a graft-versus-tumor effect from alloreactive donor T cells. Given a degree of immune incompatibility between the recipient and the donor, patients receive immunosuppression to prevent graft rejection as well as graft-versus-host-disease (GVHD), an immune-related complication of HCT [2].

Donor selection is an important aspect of optimizing the success of allogeneic transplantation. Human leukocyte antigen (HLA) identical sibling donors have historically been preferred due to their association with a lower risk of graft failure and GVHD. Patients without HLA-identical sibling donors can pursue matched or mismatched unrelated donors, and haploidentical donors (such as parents, siblings, or children) which have become increasingly successful due to advances in transplant approaches and GVHD prevention. Post-transplant cyclophosphamide has become the standard of care for GVHD prophylaxis in recipients with HLA-matched and mismatched unrelated donor allograft [1].

### HCT Timeline

The HCT process is divided into 4 distinct phases. The pre-transplantation phase consists of an assessment of a patient’s disease status and co-morbidities. Certain patients may not be deemed suitable candidates for HCT if the risks of treatment-related mortality (TRM) exceed any potential benefit they may receive from this procedure. Once a patient is deemed to be a suitable candidate for HCT, hematopoietic progenitor cells from the patient (autologous) or donor (allogeneic) are collected via apheresis to be infused into the recipient. In the transplantation phase, the patient is given a chemotherapy conditioning regimen with subsequent infusion of donor hematopoietic progenitor cells. Pre-transplant conditioning, administered before stem cell transplantation, aims to provide sufficient immunoablation to prevent graft rejection and reduce tumor burden. This regimen may include total body irradiation (TBI) and chemotherapeutic agents, categorized into high-dose (myeloablative), reduced-intensity, and nonmyeloablative regimens [5]. To mitigate TBI-related toxicity, high-dose chemotherapy-based regimens utilizing alkylating agents such as busulfan and melphalan have been developed [5].

Engraftment is the process in which donor hematopoietic stem cells can restore hematopoiesis. In the early post-transplantation phase (up to day 100 post-transplant), patients are monitored for acute chemotherapy-associated toxicity as well as complications such as infections, acute GVHD and organ impairment, as well as disease relapse. The late post-transplantation phase occurs after Day 100 of transplant and focuses on monitoring for chronic GVHD and late complications of transplantation including infections, psychosocial and quality of life impairments, late organ toxicity, and secondary malignancies. Although the risk of disease relapse decreases over time, surveillance continues with long-term follow-up.

## Cardiovascular Risk Factors and Events Associated with Transplant

Several variables in the process of HCT can be associated with an increased risk of CVD. These include pre-transplant patient factors and co-morbidities, prior chemotherapy, high-dose conditioning chemotherapy, transplant-related medications, and GVHD itself.

Patients post-HCT are at increased risk for the development of cardiovascular factors such as metabolic syndrome including individual factors of hypertension, dyslipidemia, and type 2 diabetes mellitus [5–7]. Complications such as GVHD and its treatment may predispose allogeneic HCT compared to autologous HCT to these cardiovascular factors and longer-term cardiovascular events. Subsequent development of CVD in these patients can manifest most commonly as congestive heart failure, arrhythmias, and/or ischemic coronary artery disease.

### Pre-Transplant Risk Factors

#### Patient Risk Factors

There are notable common shared risk factors between CVD and malignancy, including age, race/ethnicity, sex, lifestyle behavior, and genetic predisposition. HCT recipients often have elevated CV risk, as well as pre-existing CVD, particularly in an increasingly older and more co-morbid transplant population. The incidence of pre-HCT comorbidities such as hypertension, diabetes, and hyperlipidemia are reported to be as high as 25%, 5%, and 32% respectively [8, 9]. Pre-HCT risk factors are subsequently a strong predictor of post-HCT risk; several studies have demonstrated that conventional cardiovascular risk factors such as hypertension, obesity, dyslipidemia, and diabetes are significantly associated with increased risk of both early and late cardiac events [10–12].

#### Pre-Transplant Therapy

Direct cardiotoxicity from prior anthracycline chemotherapy exposure and radiation further predisposes patients to poor cardiovascular outcomes during and post-HC [13]. Armenian et al. demonstrated that a prior cumulative anthracycline dose ≥ 250 mg/m2, in conjunction with the presence of 2 or more comorbidities (specifically hypertension, renal insufficiency, and chronic lung disease) were significantly associated, up to a tenfold increased risk, with late cardiomyopathy and congestive heart failure [14].

Radiotherapy is also commonly used before HCT and can increase the risk of cardiac dysfunction, particularly when the heart is involved in the irradiation field. Pre-HCT exposure to chest radiation is associated with an increased risk of coronary artery disease (OR 9.5, P = 0.03) post-transplant [10, 15]. A retrospective study of long-term survivors of Hodgkin lymphoma exposed to radiation observed significant valvular defects in 42% of patients and conduction defects in 75% of patients. A peak oxygen uptake (VO2max) during exercise, a known predictor of mortality in heart failure, was significantly reduced (< 20 mL/kg/m2) in 30% of these long-term survivors. [16]

Although most data regarding pre-transplant therapy’s impact on transplant CV outcomes is with anthracyclines, it is also important to note that several novel and now commonly used agents have also been associated with cardiotoxicities. Agents such as bruton tyrosine kinase inhibitor and ibrutinib are associated with a 4.7-fold risk of atrial fibrillation and a 2.8-fold risk of hypertension [17, 18]. Proteasome inhibitors, classically part of the backbone of induction chemotherapy for multiple myeloma before transplant, have also been shown to be commonly associated with adverse cardiovascular events, with heart failure being the most common, followed by hypertension [19]. Immune checkpoint inhibitors (ICI) are increasingly used for hematologic malignancies, particularly in the relapsed/refractory setting, which may also be an indication for transplant. A recent retrospective study reported by Lin et al. observed an incidence of adverse cardiac events with ICIs of 12.5%, with the most common events being arrhythmias (9.3%), followed by myocarditis (2.1%), acute myocardial infarction (1.7%), pericarditis (1.2%), and cardiomyopathy (0.9%) [20]. The impact on subsequent post-HCT CVD outcomes, however, remains largely unknown.å

### Pre-HCT Cardiac Risk Assessment

#### Comorbidity Evaluation

The pre-transplant evaluation incorporates the HCT Comorbidity Index (HCT-CI), developed by Sorror in 2005, which scores 17 organ-specific comorbidities, including several cardiovascular (CV) conditions, to predict non-relapse mortality post-transplant [21]^.^ This tool, validated in numerous studies, includes arrhythmia, congestive heart failure (CHF), coronary artery disease (CAD), cerebrovascular disease, valvular disease, diabetes, and obesity among its CV risk factors. Recent analyses have highlighted pre-transplant diabetes and CV disease as key predictors of non-relapse mortality, emphasizing the impact of pre-existing CV conditions on transplant outcomes [21–24, 7].

#### Cardiovascular Risk Stratification

The primary goal of pre-transplant assessment is to identify high-risk CV diseases through thorough history-taking and physical examination to determine a recipient's fitness to endure the HCT process, acknowledging the potentially cardiotoxic nature of conditioning regimens, rapid volume shifts, and increased oxygen demands. Studies have shown an increased risk of cardiovascular complications at an earlier age (median age 54) among hematopoietic cell transplantation (HCT) survivors, emphasizing the critical role of pre-existing conditions, and therapeutic exposures (notably anthracyclines), in elevating this risk. [7, 25, 26]

Risk factors are categorized into non-modifiable, such as age—with associations showing a 1.6–1.8-fold increased risk of cardiac dysfunction in patients over 60 treated with anthracyclines and/or trastuzumab—and modifiable, including hypertension, diabetes, dyslipidemia, obesity, and smoking [26]. Among HCT survivors treated with high-dose anthracyclines, Armenian et. al. reported up to fivefold risk of heart failure compared to age- and sex-matched individuals from the general population and additionally increased with pre-existing conditions, hypertension, and diabetes [7].

It underscores the importance of aggressive screening and management of cardiovascular risk factors, given the observed under-treatment of conditions like hypertension, dyslipidemia, and diabetes post-transplant. The findings advocate for tailored intervention strategies, including prospective studies to assess the effectiveness of standard cardiovascular risk management in HCT survivors. The necessity of lifestyle modifications to mitigate cardiovascular morbidity is also noted, alongside the challenges in tracking late adverse effects due to survivor mobility. [27]

#### Diagnostic Tools and Treatments

Echocardiography assesses cardiac function, including ejection fraction and valvular health, but may not detect early signs of cardiovascular function and coronary artery narrowing [7]. Additional evaluations might include resting electrocardiograms, computed tomography for coronary and aortic calcifications, and more specialized tests like coronary CT angiography or myocardial perfusion testing for those at high risk [7, 25]. Additional research is required to investigate the role of standardizing cardiac evaluation.

The American Society of Clinical Oncology (ASCO) underscores the importance of comprehensive history and physical examinations for patients previously treated with cardiotoxic drugs, advocating for regular evaluation of cardiovascular risk factors such as smoking, hypertension, diabetes, dyslipidemia, and obesity [26]. Heart-healthy lifestyle modifications are encouraged, alongside echocardiograms at 6- and 12-months post-cancer therapy for asymptomatic patients at increased cardiac risk [26].

For symptomatic individuals, a shared medical decision-making approach is recommended, with options including cardiac MRI or MUGA scans if echocardiography is not feasible. Early detection of cardiac dysfunction via serum cardiac biomarkers, such as troponins and natriuretic peptides, is advised, with referrals to cardiologists for patients exhibiting cardiac dysfunction. Elevations in cardiac markers may precede findings on echocardiogram. Recommendations extend to aerobic training for disease prevention and management, given its benefits on blood pressure, lipid levels, and insulin sensitivity.

Pre-HSCT treatment focuses on optimizing current cardiac conditions and addressing reversible diseases due to prolonged survival and treatment-induced late-occurring morbidity and mortality from cardiac causes [26].

Suggested interventions to mitigate the cardiovascular risk are prophylactic therapy before and during HCT to prevent anticipated injury; primary intervention by providing therapy to treat and prevent early signs of myocardial and/or cardiac vascular damage; secondary prevention by providing therapy after the detection of LVEF decline, and tertiary treatment by providing therapy after detection of heart failure or coronary artery disease [26]. Atorvastatin is a statin that is widely used to treat hyperlipidemia and has been studied on the risk of development of cardiac dysfunction. A double-blinded randomized clinical trial demonstrated that lymphoma patients receiving anthracycline-based chemotherapy treated with atorvastatin demonstrated a reduction of the incidence of cardiac dysfunction [28]. Similarly, sodium-glucose co-transported 2 inhibitors (SGLT2) demonstrated efficacy in patients with cancer therapy-related cardiac dysfunction or heart failure by demonstrating decreased heart failure exacerbation and all-cause mortality [29]. Management of cardiac dysfunction by chemotherapy has been studied but additional studies are required particularly for transplant patients due to their unique risk factors.

Aerobic training is a cornerstone of disease prevention and treatment and decreases lipids, improves insulin sensitivity, and lowers blood pressure [7]. HSCT recipients require an average of 4 or more weeks of inpatient hospital stay which leads to muscle loss and decreased exercise capacity impacting cardiopulmonary fitness. Given the propensity of deconditioning among HSCT patients due to treatment-related complications, the American College of Sports Medicine (ACSM), recommends home-based exercise regimens, allowing recovery of the immune system before returning to gym facilities [30]. Studies indicate that initiation of exercise before and during transplantation can be safely performed [30]. Physical therapy leads to higher rates of engraftment and shorter duration of total parenteral nutrition as well as a decreased need for blood transfusions [30]. Contraindications to exercise include but not limited to are unstable angina, decompensated heart failure, ventricular arrhythmia, pulmonary hypertension, and severe valve disease but referral to cardio-oncology can result in referral to cardiac rehabilitation which is in a supervised environment [30].

### Post-Transplant Cardiac Events

Cardiac events post-hematopoietic cell transplantation (HCT) are generally categorized based on their occurrence: early (within 100 days) and late (> 100 days post-transplant). Conditioning-regimen-mediated damage to the neurohormonal system and vascular endothelium as well as immunological and inflammatory effects of allografting predispose to the development of cardiovascular disease. Atrial fibrillation has been highlighted as a major post-transplant concern, with studies by Tonorezos et al. and Chang et al. illustrating its association with increased mortality risk [22, 31, 32]^.^ Additional complications include pericarditis, dyslipidemia, type 2 diabetes mellitus, valvular heart disease, and right ventricular systolic dysfunction, underscoring the need for vigilant cardiovascular monitoring and intervention [33–39]^.^

Graft-versus-host disease (GVHD) has been linked to the development of cardiovascular risk factors such as hypertension, diabetes, and dyslipidemia. Rackley et al. identified acute GVHD as independently associated with hypercholesterolemia and hypertriglyceridemia post-allogeneic transplant [40]. Its inflammatory nature also correlates with increased thrombosis risk and potential microvascular disease exacerbation [41]. Pharmacologic therapy for the prevention and treatment of GVHD may also have an impact on cardiovascular complications. GVHD prevention standards include steroids, calcineurin inhibitors (tacrolimus/cyclosporine), sirolimus, and methotrexate or mycophenolate. Sirolimus may elevate hypertriglyceridemia and hyperlipidemia risk, while mycophenolate mofetil (MMF) and corticosteroids have been linked to hyperlipidemia [42]. High-dose post-transplant cyclophosphamide (PT-Cy) has also emerged as an effective GVHD prevention strategy [41], but also has been associated with cardiotoxicity, although data can be conflicting. Medications like ruxolitinib and ibrutinib used to treat acute and chronic GVHD have been associated with a 4.7–5.8-fold increase in atrial fibrillation risk and a 2.8-fold increase in hypertension and hyperlipidemia risk, respectively [41].

PT-Cy use decreases the incidence of GVHD but has been suggested to be associated with cardiac events within the first 100 days post-transplantation, negatively impacting overall survival. Historically, Cy-related acute cardiotoxicity, which includes endothelial injury, arrhythmias, and heart failure, often occurs within 10 days of administration. Dulery et al. found that the incidence of early cardiac events was significantly higher in patients receiving post-transplant cyclophosphamide (PT-Cy) at 19%, compared to 6% in those who did not [37]. These events encompassed left ventricular systolic dysfunction, acute pulmonary edema, pericarditis, arrhythmias, and acute coronary syndrome. Risk factors for increased early cardiac event incidence included PT-Cy administration, advanced age, a sequential conditioning regimen, and prior cyclophosphamide exposure before HCT37 [37, 38]. Additional data is needed to further understand the potential increased cardiotoxicity with PT-Cy but underscores the importance of careful monitoring with PT-Cy in higher-risk patients.

Studies investigating PT-Cy-associated cardiac toxicity illustrate the challenges in evaluating and mitigating cardiac risk among stem cell transplant recipients [39, 40]. Two studies by Yeh et al. and Perez-Valencia et al. investigated the cardiac toxicities following allogeneic HCT and the use of PT-Cy for GVHD prophylaxis [38, 39]. Yeh et. al., studied 585 patients and found a 6.5% incidence of cardiac toxicities post-allo-HCT, slightly higher in PT-Cy recipients (7.4%) compared to non-PT-Cy recipients (5.8%) [39]. Significant predictors of cardiac toxicity included age over 55 and pre-existing conditions such as hypertension and diabetes. This toxicity correlated with poorer one-year survival outcomes, although PT-Cy use was also linked to some improvement in outcome as well [39, 40]. The Perez-Valencia et al. study reported an 8.4% incidence of early cardiac events (ECEs) within 180 days post-transplant, with a higher incidence in patients receiving PT-Cy, particularly when combined with TBI [38]. ECEs, including heart failure and pericardial complications, were associated with higher mortality and lower survival rates. Both studies highlight the importance of cardiac risk assessment and monitoring in patients undergoing allo-HCT with PT-Cy, especially when combined with TBI [38, 39].

#### Early Cardiac Events

Atrial fibrillation and atrial flutter are the most common cardiac events occurring within the first 100 days of HCT, with an estimated incidence of 2–10% [14–18]. Congestive heart failure has also been reported as a predominant early post-transplant complication with an incidence of 2.2% [43–45]. While arrhythmias are associated with longer-term 1-year mortality, acute cardiovascular death from any cause is rare [24]. Other cardiovascular events such as pericardial effusion, pericarditis, and myocarditis are also uncommon (< 1%) but are associated with inferior survival [26, 46, 47].

Several studies have demonstrated risk factors associated with early cardiovascular events [26, 46, 47]. Patients with an ejection fraction of less than 50% had 5.3 higher odds of a cardiovascular event within the first 100 days than patients with an ejection fraction of > 60% (p = 0.02) [43]. While patients aged ≥ 55, hypertension and a history of cardiovascular disease, arrhythmia, and diabetes were noted to be significant predictors of the development of cardiac toxicity by day 100 after allogeneic HCT; of note, the cardiac toxicity did not significantly vary between patients receiving PT-Cy and non-PT-Cy GVHD prophylaxis [39]. The underlying pathophysiology for early cardiac complications is multifactorial but can be attributed to inflammation, oxidative stress, calcium homeostasis alteration, and programmed cell death mechanisms which lead to endothelial dysfunction, myocardial damage, apoptosis, and calcium overload [48]. Specifically in allogeneic HCT, the release of proinflammatory cytokines in GVHD can lead to leukocyte activation, endothelial injury, and vascular leakage which can lead to cardiac complications such as arrhythmias as well as the rare occurrences of pericardial effusion and pericarditis [48, 49].

The incidence of short-term cardiovascular events was similar between myeloablative, reduced intensity, and nonmyeloablative conditioning regimens among allogenic recipients [26]. Prior exposure to anthracycline of 250 mg/m^2^ was associated with increased short-term cardiovascular events among autologous recipients compared to allogenic recipients [26].

#### Late Cardiac Effects

Advancements in transplantation and supportive care have led to an increase in long-term HCT survivors but also revealed late adverse effects, notably cardiovascular disease, and metabolic syndrome. The leading causes of non-disease-related mortality among HCT survivors are solid tumor malignancies and cardiopulmonary diseases [50]. In the Bone Marrow Transplant Survivor Study, the risk of premature cardiovascular-related death following HCT was 2.3-fold for allogenic HCT recipients and 1.3 for autologous HSCT recipients compared to the general population [47, 49]. In a retrospective cohort study of both autologous and allogeneic HCT recipients compared to age-matched controls from the general population by Chow et al., several risk factors for increased risk of cardiovascular death were identified, including age ≤ 60 years at the time of transplant, mobilized peripheral blood stem cells, relapse of the primary disease, as well as the presence of multiple risk factors, (e.g. hypertension, dyslipidemia, diabetes, and renal disease) [49]. HCT recipients had greater all-cause mortality compared to the general population comparator group with an incidence rate difference of 39.1 (95% CI, 33.7–44.6) and cardiovascular death with an incidence rate difference 3.6 (95% CI 1.7–55) [49].

The prevalence of cardiovascular risk factors was also found to be significantly higher among HCT patients compared with the general population in a study by Armenian et al., as such that 10 years post-HCT the incidence of hypertension was 37.7%, diabetes 18.1%, and dyslipidemia 46.7% [47]. De-novo development of hyperlipidemia and hypertriglyceridemia following allo-HCT was estimated at 43–73% [47]. This is further pronounced with pre-existing cardiovascular risk factors, significantly elevating the risk of CVD, with a five-fold increase for a single factor and a 20-fold increase for multiple factors [51] Even controlling for age, cardiotoxic therapeutic exposures, and stem cell source, patients with multiple cardiovascular risk factors were at 1.5-fold higher risk of developing CVD [41, 52]. TBI has also been reported as an increased risk of developing dyslipidemia, diabetes, and hypertension [49]. Armenian et. al., estimated the risk of coronary disease increased by 9.5 fold with the administration of chest radiation prior to transplant [10]. Additionally, HCT is associated with an increased risk of chronic kidney disease which is also an independent risk factor for cardiovascular disease [49].

Allogenic recipients are at about 2 times greater risk of developing long-term cardiovascular complications compared to autologous HCT patients [26]. Interestingly, a pretransplant left ventricular ejection fraction of < 50% and history of heart failure was associated with a higher risk of late cardiac events among autologous recipients but not among allogenic HCT patients [26], while overall 10-year incidence of heart failure was similar. In another observational study of 1244 autologous HCT recipients, the cumulative incidence of congestive heart failure was 4.8% at 5 years post-transplant which further increased to 9.1% at 15 years post-transplantation, equating to a 4.5-fold higher risk of heart failure among autologous HCT recipients compared to the general population [52].

The late cardiac effects of HCT have gained significant research interest, notably concerning the development of dyslipidemia, glucose intolerance, and hypertension following immunosuppressive therapy and total body irradiation, although the underlying mechanisms remain unclear [51].

Immunosuppressive agents, such as calcineurin inhibitors and corticosteroids, used in the treatment of GVHD are well-recognized risk factors and withdrawal of these medications does not lead to the resolution of cardiovascular disease [51]. More novel agents such as ibrutinib and ruxolitnib have also been associated with increased risk of hyperlipidemia and arrythmias [52–54]. Patients with grade II-IV GVHD were at the highest risk of developing cardiovascular disease and diabetes at 10 years after HCT [51]. The underlying etiology is attributed to the highly inflammatory state which leads to vascular injury and accelerated atherogenesis.

### Post HCT Cardiac Risk Assessment and Management

Long-term survivors after hematopoietic cell transplantation (HCT) face a significant.

increased risk, ranging between 31 to 49% of developing metabolic syndrome and cardiovascular disease at a younger age [15]. The median age of the first cardiovascular event ranges between 35–66 years of age (median 53 years) which is significantly lower compared to the general population (median age 67 years) [15]. This risk is particularly higher among recipients of allogeneic HCT due to the higher incidence of GVHD and the complications arising from its treatment. General risk factors influencing these assessments include the type of transplant (allogeneic or autologous), the use of TBI as part of pre-transplant conditioning, the development of acute or chronic GVHD, and ongoing therapy with corticosteroids, calcineurin inhibitors, or sirolimus. Additionally, the presence of other metabolic syndrome components post-HCT further complicates cardiac risk assessment and management. It is important to consider that the incidence of CVD increases over time and the presence or absence at initial evaluation pre- or post-transplant does not predict long-term cardiac toxicities [55]. A comprehensive analysis of the available information including laboratory workup and imaging is recommended.

An international group of experts has updated the screening and preventive practices for long-term HCT survivors [55]. As such, current guidelines recommend annual screening for cardiovascular risk factors, with specific considerations for HCT patients largely informed by retrospective studies [7, 51, 56]. Metabolic syndrome encompasses risk factors for both diabetes and cardiovascular disease and is associated with increased all-cause mortality [55]. It is estimated that 31–53% of HCT survivors meet the diagnostic criteria, visceral obesity, elevated blood pressure, hyperglycemia, high triglyceride, or low high-density lipoprotein [50, 55].

It is recommended to measure blood pressure and weight and consider the patient’s personal and family history, lifestyle factors, type of transplant (autologous vs. allogeneic), cumulative anthracycline dose, history of chest radiotherapy, TBI, chronic CNI use, treatment of GVHD with sirolimus or glucocorticoids [55]. Further surveillance, prevention, and treatment should be tailored according to those individuals on every visit [55].

Hypertension should be assessed at every clinic visit, with anti-hypertensive medication initiation guided by the Joint National Committee guidelines [55]. Lipid assessment at 6 months post-transplant and annually if low-risk factors and high-risk or ongoing use of glucocorticoids, CNI, or sirolimus, every 3–6 months is recommended. Treatment should follow the AHA/ACC recommendations.

Diabetes screening in general is advised every three years, but among HCT survivors, it is recommended to screen at 3–6 months post-HCT. More frequent screenings with HbA1c with ongoing risk factors such as corticosteroid therapy every 3–6 months thereafter are recommended. And in low risk for cardiovascular disease annual screening after initial screening is recommended [55]. Both lifestyle and pharmacotherapy interventions are essential.

Obesity is a critical factor that exacerbates the risk of hypertension, dyslipidemia, type II diabetes, coronary heart disease, and ischemic stroke. Although body mass index (BMI) is commonly used, waist circumference provides a more accurate risk assessment by indicating visceral adipose deposits, an independent risk factor for cardiometabolic health. HCT survivors, particularly those treated with corticosteroids for GVHD, are at risk for sarcopenic obesity [55]. Despite the lack of specific guidelines for screening abdominal obesity in HCT survivors, promoting regular exercise, maintaining a healthy weight, and dietary counseling are key. For individuals with a BMI ≥ 30 kg/m^2^ or waist circumference > 40 inches in men and > 35 inches in women, intensive, multicomponent behavioral interventions are recommended. Dual-X-ray absorptiometry can aid in evaluating and monitoring body composition changes.

HCT patients also have an elevated risk of developing dyslipidemia. Early assessment of exposures and risk factors is crucial, including family and personal history, obesity, high-dose TBI, grades II-IV acute GVHD, chronic GVHD, chronic liver disease, and immunosuppressant therapy. Multimodal weight loss treatment for obese patients is recommended.

## Future Directions

Recent studies have emphasized the predictive value of cardiac biomarkers and imaging in assessing CV disease risk post-HCT. The combination of high-sensitivity cardiac troponin T (hs-cTnT) and N-terminal pro-B-type natriuretic peptide (NT-proBNP) has shown superiority in predicting cardiac events over individual measurements [57]. While the biomarkers are useful in symptomatic patients, given the inter-patient variability among asymptomatic patients, routine measurements are not indicated. Additionally, global longitudinal strain (GLS) analysis offers insights into cardiovascular outcomes, highlighting the significance of long-term cardioprotective medication adherence [58]. Future studies are required to evaluate the predictive value of blood biomarkers and echocardiographic findings [15].

Additionally, risk stratification tools, such as the Framingham risk score (FRS) and the AHA/American College of Cardiology risk calculation online tool, are commonly used to risk stratify patients. However, FRS is derived from the general population to determine the benefit of treatment with anti-hypertensives and lipid-lowering agents and demonstrated shortcomings for the HCT patients. Cardiac computed tomography, coronary CT angiography, and coronary calcium score have demonstrated increased sensitivity, and positive and negative predictive value compared to the traditional FRS [59]. Armenian et. Al. developed a validated, risk prediction model that identifies overall CVD based on age, anthracycline dose, chest radiation, hypertension, diabetes, and smoking [15]. It identifies low-, intermediate, and high-risk groups corresponding to a cumulative 10-year incidence of CVD and a framework to modify the screening and intervention to reduce the risk of CVD after HCT [15]. Gangaraju et al. also created a risk prediction nomogram to predict CVD at 10 and 20 years after the transplant, including factors such as sociodemographic, smoking, diabetes, hypertension, arrythmia and chest radiation [57]. This model proposes to inform targeted screening for early detection and guide treatment on risk factor control [53]. Additional research studies such as clinical trials evaluating the role of screening and type management are critical given the advancements within cardiology and the unique challenges that HCT survivors face [57]. Furthermore, the use of artificial intelligence tools can further assist.

Against this backdrop of evolving risk stratification methods, the significance of understanding genetic predispositions to cardiovascular diseases post-HCT is underscored. Clonal hematopoiesis (CHIP), characterized by the expansion of hematopoietic stem cell clones due to somatic mutations, mosaic chromosomal alterations, and copy number variants, is increasingly recognized for its role in adverse cardiovascular outcomes in patients undergoing hematopoietic cell transplantation. CHIP is associated with aging-related health conditions such as coronary artery disease, heart failure, and stroke [60]. Studies have demonstrated that CHIP, particularly mutations in TP53 and TET2, is associated with heightened myocardial toxicity from anthracycline chemotherapy, attributing to enhanced neutrophil migration to injured myocardial tissue [61]. Furthermore, research by Rhee et al. has shown that in multiple myeloma patients with CHIP undergoing HCT, there is a significant increase in the incidence of cardiovascular diseases post-transplant, underscoring the prognostic value of pre-HCT CHIP as a potential biomarker for cardiovascular risk assessment [60].

Gibson et al.’s investigation into the whole-genome sequencing pre- and post-autologous stem cell transplantation revealed that patients with CHIP exhibit notably inferior overall survival rates, including an elevated risk of death from transplant-related mortality and cardiovascular disease [61]. This body of research emphasizes the critical impact of CHIP on transplant outcomes and the necessity for comprehensive cardiovascular risk management in the pre-transplant assessment, highlighting the complex interplay between genetic predispositions, somatic mutations, and cardiovascular health in the HCT population.

Building on the understanding of genetic and cardiovascular risk factors, the focus shifts towards addressing another significant challenge facing HCT survivors: sarcopenia. It is defined as the loss of skeletal muscle mass, strength, and function. Sarcopenia in HCT survivors is linked to increased mortality risk, underscoring the need for interventions to boost muscle mass, metabolism, and strength. Song et. Al. aims to combat sarcopenia (muscle loss) in adolescents and young adults (AYAs) who have had hematopoietic cell transplantation (HCT), a group at high risk of this condition due to the impacts of HCT on the musculoskeletal system [62]. The research will test a 16-week program of aerobic and resistance exercise combined with nicotinamide riboside (NR), a supplement that enhances muscle energy production, in a randomized controlled trial involving 80 AYAs post-HCT [62]. The study aims to evaluate the effects on muscle strength, cardiovascular fitness, muscle mass, and energy metabolism, comparing exercise plus NR, exercise alone, NR alone, and a control group [62]. The goal is to determine whether exercise combined with NR more effectively improves muscle health than exercise alone, with findings expected to inform tailored strategies to reduce chronic disease in cancer survivors [62].

## Conclusion

This review highlights the complex interplay between hematopoietic cell transplantation (HCT) and cardiovascular health, emphasizing the multifaceted cardiovascular risks that stem from pre-transplant patient factors, treatment regimens, and post-transplant complications. With the growing population of HCT survivors, it's imperative to integrate comprehensive cardiovascular risk assessments and management strategies throughout the HCT process. This review underscores the imperative need for continued research and development in the field of cardiovascular and musculoskeletal health among HCT survivors. By integrating findings from recent studies on cardiac biomarkers, genetic predispositions, and interventions for sarcopenia, a clearer picture emerges of the multifaceted challenges these patients face. Future research should focus on bridging the current gaps, leveraging advancements in biomarker discovery, risk stratification methodologies, and the potential of artificial intelligence, to foster a holistic approach towards managing and mitigating these risks. The evolution of personalized medicine in this context holds the promise of significantly improving the quality of life and survival outcomes for individuals undergoing hematopoietic cell transplantation.

## Key References

- Bhatia S, et al. Trends in Late Mortality and Life Expectancy After Allogeneic Blood or Marrow Transplantation Over 4 Decades: A Blood or Marrow Transplant Survivor Study Report. JAMA Oncol. 2021;7(11):1626-1634.
  - This reference is of major importance because it is the largest study of long-term survivors of allogeneic hematopoietic cell transplant, demonstrating that late mortality has declined over time, but life expectancy is not restored to expected rates of the general US population. It also demonstrates leading causes of late mortality including infection, subsequent malignant neoplasms and cardiovascular disease.

- Vasbinder A, Hoeger CW, Catalan T, et al. Cardiovascular Events After Hematopoietic Stem Cell Transplant: Incidence and Risk Factors. JACC CardioOncol. 2023;5(6):821–832.
  - This reference is of importance as it is one of the largest cohort describing the incidence and risk factors for short- and long-term cardiovascular events in a contemporary cohort of hematopoietic cell transplant recipients.

- Rotz SJ, Bhatt NS, Hamilton BK, et al. International Recommendations for Screening and Preventative Practices for Long-Term Survivors of Transplantation and Cellular Therapy: A 2023 Update. Transplant Cell Ther. 2024.
  - This reference is of major importance as this is the most recent updated international guidelines for screening practices for long-term survivors of hematopoietic cell transplantation.

- Gangaraju R, Chen Y, Hagerman L, et al. Prediction of Coronary Heart Disease Events in Blood or Marrow Transplantation Recipients. JACC: CardioOncology. 2023; 5(4):504-517.
  - This reference is of importance as this study developed a cardiovascular disease risk prediction nomogram using a large cohort of hematopoietic cell transplant survivors and factors including sociodemographics, comorbidities and therapeutic exposures.

- Rhee JW, Pillai R, He T, et al. Clonal Hematopoiesis and Cardiovascular Disease in Patients With Multiple Myeloma Undergoing Hematopoietic Cell Transplant. JAMA Cardiol. 2024;9(1):16-24.
  - This reference is of importance as it highlights the emerging importance and association of clonal hematopoiesis with late cardiovascular disease in survivors of hematopoietic cell transplantation.
